# Stress Corrosion Cracking (SCC) Resistance of the AW-5083 Alloy with a Plasma Electrolytic Oxidation (PEO) Coating in the Presence of Chloride (Cl^−^)

**DOI:** 10.3390/ma19010039

**Published:** 2025-12-22

**Authors:** Grzegorz Hajdukiewicz, Aleksander I. Komarov, Kamil Jurczak, Dmitry V. Orda

**Affiliations:** 1 Faculty of Marine Engineering, Gdynia Maritime University, 81-225 Gdynia, Poland; 2 Joint Institute of Mechanical Engineering of the National Academy of Sciences of Belarus, The State Scientific Institution, 220072 Minsk, Belarus; al_kom@tut.by (A.I.K.); dmitry_orda@mail.ru (D.V.O.); 3 Mechanical and Electrical Engineering Department, Polish Naval Academy, 81-103 Gdynia, Poland; k.jurczak@amw.gdynia.pl

**Keywords:** stress corrosion cracking (SCC), AW-5083, plasma electrolytic oxidation (PEO)

## Abstract

This article presents a comparative study of mechanical properties and stress corrosion cracking (SCC) resistance of bare AW-5083 aluminum alloy and the same alloy coated by plasma electrolytic oxidation (PEO). Although Al–Mg alloys of the 5XXX series have been extensively studied with respect to SCC behavior, data concerning their performance after PEO treatment under mechanical loading in chloride-containing environments remain scarce. Prior to SCC testing, potentiodynamic polarization measurements were performed to assess the barrier properties of the PEO coating against general corrosion. The results demonstrate that the PEO coating significantly modifies the electrochemical response of the alloy and improves its resistance to corrosion processes in the presence of chloride ions. SCC tests revealed that the application of the PEO coating leads to enhanced resistance to stress-assisted degradation of the AW-5083 alloy, while distinct features of coating cracking under tensile loading were observed and discussed. The study provides new experimental insight into the combined mechanical and electrochemical behavior of PEO-coated AW-5083 alloy exposed to chloride environments.

## 1. Introduction

Aluminum and its alloys are characterized by a unique and attractive combination of physical and mechanical properties, such as relatively high elastic modulus (E) and low density (*ρ*), which makes them one of the fundamental material groups in transportation engineering and low-weight construction. Since the beginning of aluminum alloy production, continuous efforts have been made to improve their performance, including refining mechanical and heat treatment routes in order to increase fatigue and corrosion–fatigue strength as well as overall durability [1,2,3,4,5].

The next logical step in improving aluminum alloy performance is to enhance their surface properties, for example, through oxidation. For many years, oxide coatings formed by anodic oxidation have been extensively used to improve the surface performance of aluminum alloys in architectural, mechanical, transportation, and medical applications. Such oxide layers are characterized by high hardness and excellent resistance to corrosion and tribological wear. The porous structure of anodic oxide layers facilitates effective dyeing, enabling the production of various aesthetically pleasing products [1,2,6]. Furthermore, Plasma Electrolytic Oxidation (PEO) coatings exhibit significantly stronger adhesion to the substrate than conventional protective coatings, such as technical or paint coatings [1,7,8].

In industrial practice, oxidation techniques using acidic electrolyte solutions are commonly applied, and several of these processes have been standardized over time. Another process, in which aluminum alloys are coated with an Al_2_O_3_ layer in an alkaline electrolyte, was developed and independently patented in the 1970s in the United States and the Soviet Union and later found broad industrial application. This method is known as Plasma Electrolytic Oxidation (PEO), also referred to as Micro-Arc Oxidation (MAO) [9], Micro-Arc Discharge Oxidation (MDO), Spark Anodizing, Micro-Plasma Oxidation, or Anodic Spark Deposition (ASD) [10]. Numerous studies have reported that PEO coatings exhibit greater thickness [11,12], lower porosity [12], higher hardness, and improved tribological performance compared with conventional anodic layers [11,12,13,14].

Aluminum–magnesium alloys, including the AW-5083 alloy, belong to the group of non-heat-treatable materials, which are known for their good corrosion resistance but limited resistance to stress corrosion cracking (SCC) [15,16]. This alloy is widely used in maritime [17], aviation [18], and the automotive industry [19], and even in medicine, particularly implantology [20], as it combines favorable mechanical properties with relatively high resistance to general corrosion. Al–Mg alloys are frequently exposed to chloride-containing environments due to their widespread application in marine structures, shipbuilding, offshore constructions, and coastal infrastructure [17], as well as in the automotive sector through contact with chloride-based de-icing agents used for winter road maintenance. [16]. Nevertheless, their susceptibility [15] to SCC in the presence of chloride ions significantly limits their long-term service durability, primarily due to the presence of the anodic β phase (Al_3_Mg_2_) precipitating along grain boundaries, as widely reported in the literature for sensitized Al–Mg alloys [1,21,22]. This phase is known to promote the initiation of localized corrosion, accelerate dealloying processes and, under appropriate conditions, favor an intergranular fracture mode. These phenomena are of particular relevance to the authors of this study, who routinely analyze material degradation in structures operated in marine conditions, where prolonged chloride exposure under sustained stress represents one of the most critical operational hazards. In order to improve corrosion resistance of the aluminum alloys, the novel methods for surface modifications such as PEO are increasingly applied [23]. PEO enables the creation of a complex, multi-layer oxide coating that consists mostly of Al_2_O_3_ which is characterized by high hardness, high adhesion to a substrate, and resistance to tribological wear [11,12]. PEO coatings have also found industrial application in the automotive and aerospace sectors, where they are used to improve the wear and corrosion resistance of aluminum components operating under demanding service conditions [16].

Despite the growing interest in PEO technology in the context of corrosion resistance [23,24,25], the available literature lacks a detailed analysis of its effectiveness in protecting aluminum alloys, in particular the AW-5083 alloy, against stress-corrosion cracking (SCC). Only limited attention has been paid to direct comparisons of corrosion behavior between uncoated aluminum alloys and those with PEO coatings. Therefore, this study seeks to address this gap by examining the influence of Al_2_O_3_ PEO coatings on the corrosion resistance of the AW-5083 aluminum alloy under chloride-induced SCC conditions at a constant tensile stress equal to 80% of R_p0.2_ (conventional yield strength, extensometric plastic strain, where 0.2 denotes the conventional elongation value expressed in percent according to PN-EN ISO 6892-1:2020-05). To achieve this goal, potentiodynamic polarization measurements and SCC tests under constant tensile stress were conducted on PEO-coated and uncoated specimens of the AW-5083 aluminum alloy.

## 2. Materials and Methods

### 2.1. Characteristics of the Material and Preparation of the Samples

**Figure 1:**
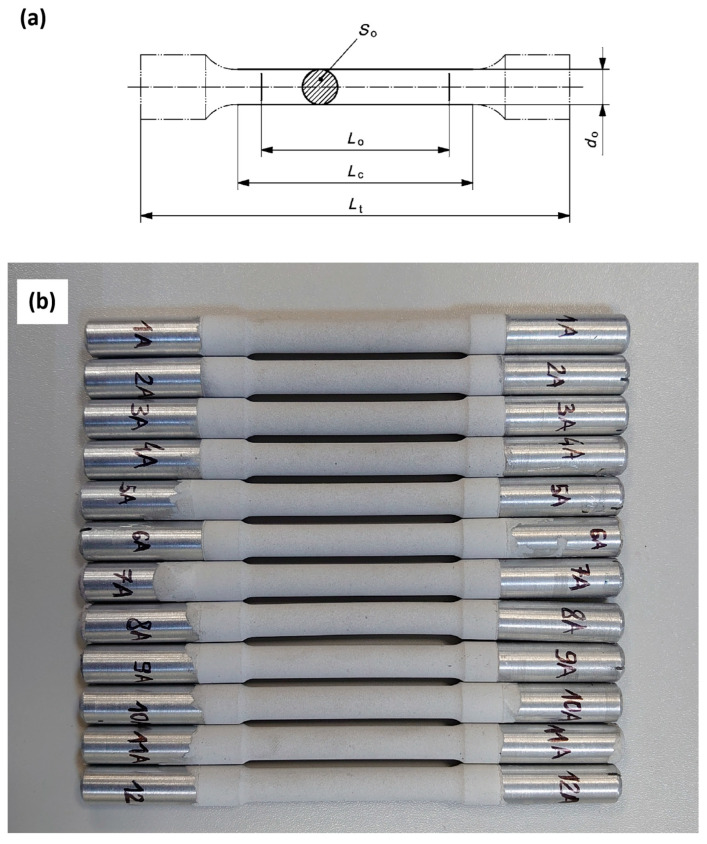
(**a**) Geometry and dimensions of the samples for tensile testing in accordance with PN-EN ISO 6892-1 standard L_o_ = 50 mm, L_c_ = 70 mm, L_t_ = 165 mm, d_o_ = 8 mm, S_0_ = 50.27 mm^2^ (d_o_ = 5 mm for the samples used in SCC testing with threaded grip sections—Figure 2); (**b**) A photo of the samples after PEO coating.

The 18 samples used for static tensile testing were produced from a cold-rolled AW-5083 aluminum alloy sheet in the process of machining. Chemical composition of the sheet was studied with emission spectroscope, Solaris CCD Plus (Table 1). The samples were prepared in accordance with the PN-EN ISO 6892-1 standard [26] in such a way that their axes of symmetry aligned with the direction of rolling of the alloy sheet—Figure 1.

### 2.2. PEO Coating Production

Next, the 18 samples were divided into two groups. The first group consisted of nine samples in the same state as at the time of delivery without any additional treatment. The nine samples from the second group were PEO coated. The PEO process was conducted in the Joint Institute of Mechanical Engineering of the National Academy of Sciences of Belarus. The coating station consisted of a power supply and control module (with thyristor power controller that ensured adequate anode–cathode polarization of the samples) and a technological module. The substrate-to-coating conversion method involved placing the samples in the slightly alkaline electrolyte solution and then exposing them to electrical impulses up to 600 V of amplitude and frequency between 50 and 60 Hz. The samples were exposed to both anodic and cathodic polarization, with the former being the majority. The process included the following steps:

The samples from the second group were degreased with 70% ethanol (C_2_H_5_OH) solution.

1. Next, they were PEO coated in the electrolyte solution consisting of 2 g KOH and 5 g Na_2_SiO_3_ per liter of distilled water.
2. The parameters of the process were decided based on the total surface area of the submerged samples, initially set to U = 290 V, I = 40 A (30.8 A/dm^2^). The process parameters were established after their quality had been previously confirmed by microscopic examination of the metallographic cross-sections.
3. After the initial phase (5 min), the parameters were stabilized at U = 280 V, I = 35 A (26.9 A/dm^2^).
4. The process of coating lasted for 90 min for each input.

**Figure 2:**
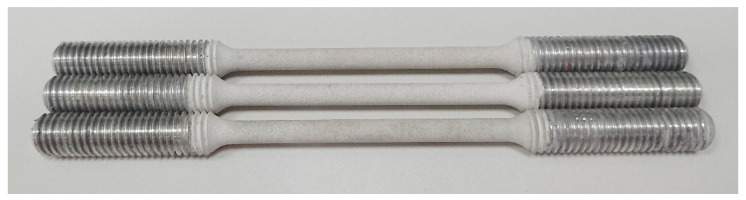
AW-5083 alloy samples with Al_2_O_3_ PEO coating and with threaded grip sections for SCC testing.

Figure 2 shows the AW-5083 alloy samples with Al_2_O_3_ PEO coating and with threaded grip sections for SCC testing. The surfaces of the samples coated with the PEO layer were not polished and were left in the as-obtained state after removal from the electrolyzer. The measured surface roughness values were Ra = 4.171 μm and Rz = 20.756 μm. Surface roughness was determined using an Olympus OLS40-SU confocal microscope (Olympus, Tokyo, Japan) equipped with LEXT OLS4100 software, version 3.1.7.14.

### 2.3. Determination of the PEO Coating Properties

**Figure 3:**
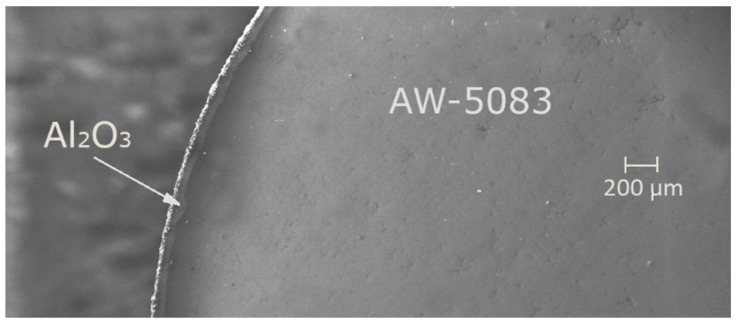
A cross-section of the PEO coated AW-5083 alloy, electron microscope Zeiss EVO MA15 (Carl Zeiss AG, Oberkochen, Germany), magnified 61×.

**Figure 4:**
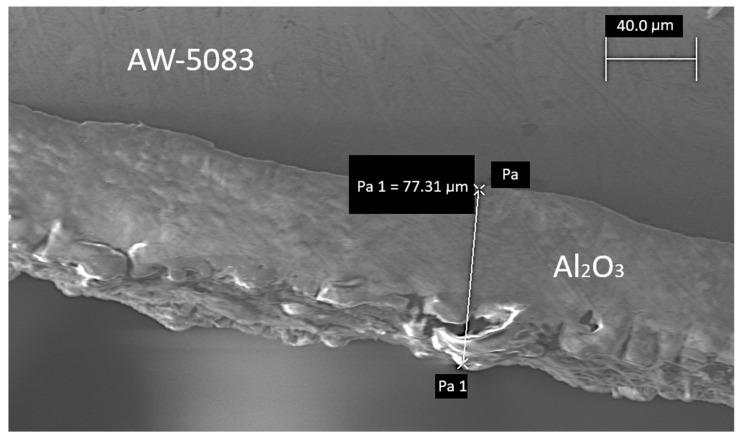
A cross-section of the PEO coated AW-5083 alloy, electron microscope Zeiss EVO MA15, magnified 921× (Pa and Pa 1 are auxiliary text for marking dimensions in the microscope software).

Afterwards, one of the samples was randomly chosen and cross-sectioned in order to determine the thickness of the coating (Figure 3 and Figure 4). This parameter fell within the range of 60 to 80 μm.

**Figure 5:**
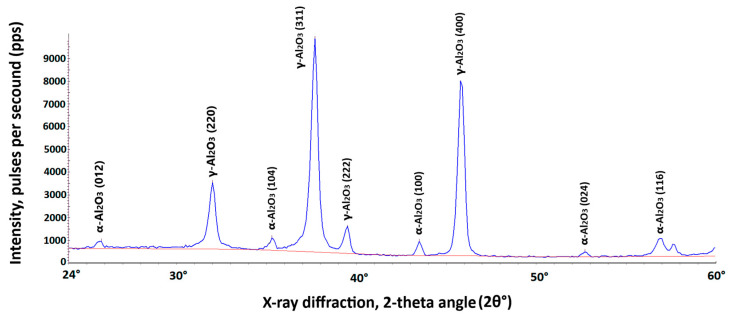
X-ray diffraction analysis of the AW-5083 alloy coating for the diffraction angle 2θ between 24° and 60° using X-ray diffractometer DRON-3M software DifWin ver. 1. The blue curve represents the diffraction spectrum of the tested sample. The red curve is the background spectrum.

Furthermore, a fragment from the same randomly selected sample was used to determine the percentage of the main aluminum oxide polymorphs on the coating’s surface. For this purpose, X-ray diffractometer DRON-3M was used. It utilizes copper anode lamp as a source of CuKα radiation that is produced at the voltage of 27 kV and 12 mA of current. Scanning is conducted for the diffraction angles 2θ between 24° and 65° with 0.1° step and exposition time of 15 s at every point (Figure 5).

Percentage of the total volume of different Al_2_O_3_ polymorphs on AW-5083 alloy coating surface was determined using the software of the DRON-3M X-ray diffractometer and amounted to, respectively,

- α-Al_2_O_3_ ~ 5.68%;
- γ-Al_2_O_3_ ~ 94.32%.

The distribution of Al_2_O_3_ polymorphs within the coating was determined directly on the surface, without any destructive sample preparation such as cutting or abrasion. The phase composition of the oxide layer typically depends on the substrate alloy, electrolyte composition, and the parameters of the PEO process [27,28]. In this study, all samples were prepared from the same aluminum sheet, and all PEO coatings were produced under identical processing conditions and in the same electrolyte solution. Therefore, the resulting coatings were considered comparable in composition and structure.

### 2.4. Potentiodynamic Testing

Prior to the SCC experiments, potentiodynamic tests (using Tafel plots) were performed on stress-free AW-5083 alloy specimens cut from the same aluminum sheet as the SCC samples. The purpose of these measurements was to evaluate the protective efficiency of the Al_2_O_3_ coating produced by the PEO process by comparing the electrochemical behavior of coated and uncoated specimens. The tests were conducted using an Autolab PGSTAT302N potentiostat with Metrohm’s NOVA 2.4.1 software in 3.5 wt.% NaCl solution at 20 °C. The electrochemical cell was configured in a standard three-electrode setup, employing a saturated calomel electrode (SCE, EK101) as the reference, a platinum mesh as the counter (auxiliary) electrode, and the tested sample as the working electrode.

### 2.5. Stress Corrosion Cracking (SCC) Testing

The research was planned and conducted as a comparative study between uncoated AW-5083 aluminum alloy and PEO coated AW-5083 aluminum alloy.

All of the samples (18 items) were divided into two groups, nine samples each. The samples from the first group were untreated, while the remaining nine samples were PEO coated (as described in the Section 2). Next, in both groups, the samples were separated into three subgroups and then grouped back in such a way that each group consisted of three untreated and three coated samples:

- The first group was used for static tensile testing–the mean R_p0.2_ value was calculated to be 146 MPa;
- The second group was used for SCC testing in 3.5 wt.% NaCl solution, at 20 °C and at the constant tensile stress value equal to 80% of R_p0.2_ (116.8 MPa) for the duration of 1500 h, after which static tensile testing was performed;
- The third group was exposed to 3.5 wt.% NaCl solution, at 20 °C for the duration of 1500 h without tensile stress, after which static tensile testing was performed.

The samples from the second and third group (the ones exposed to corrosive conditions) were weighed with 0.0001 g accuracy before placement in the 3.5 wt.% NaCl solution.

**Figure 6:**
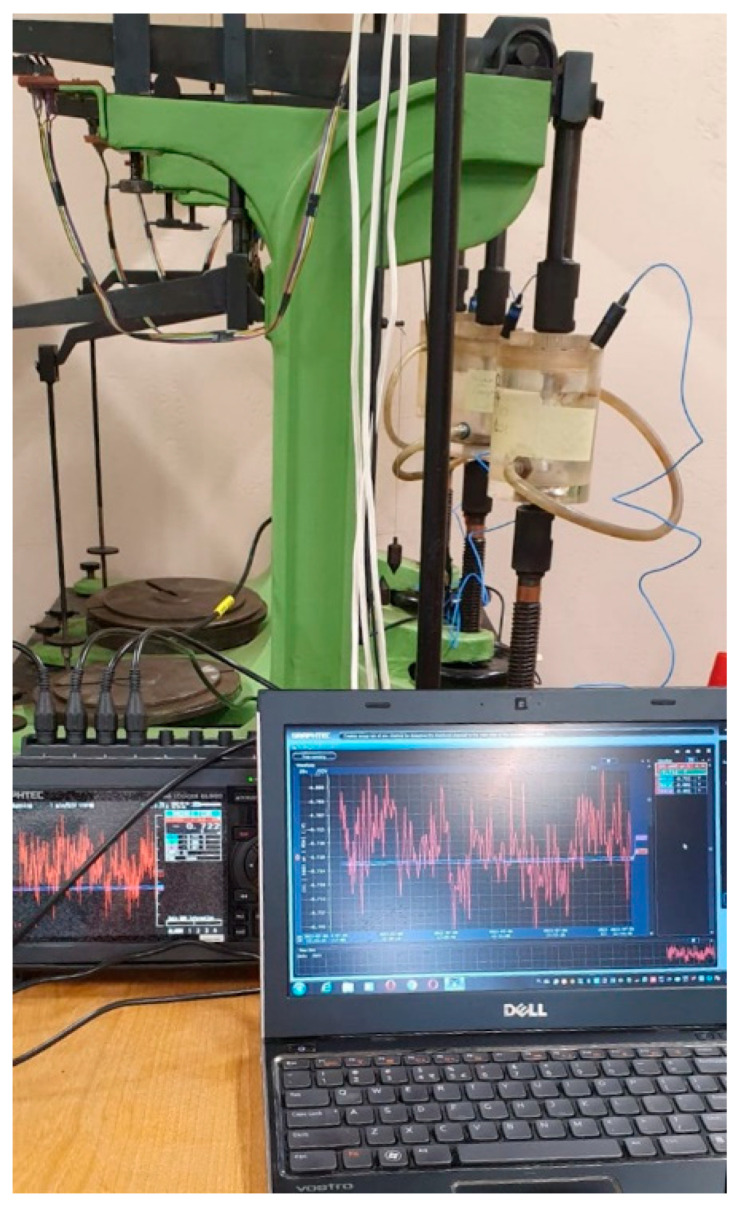
The measuring station for SCC at the constant tensile stress that can assess corrosion potential.

Figure 6 shows the measuring station for SCC at the constant tensile stress that can assess corrosion potential. The tensioning device exerts tensile stress on a given part of the sample which is placed in a container with 20 °C 3.5 wt.% NaCl solution and equipped with a saturated calomel electrode as a reference electrode. A tested sample was a counter electrode and together with the reference electrode EK-101 they were connected to a multi-channel potential measuring device working on-line. The samples were placed in the containers with NaCl solution in such a way that only the parts with PEO coating were submerged. Threaded parts (without coating) were isolated with silicone.

The initial corrosion potential *E_corr_* of the Al_2_O_3_-coated samples, measured after immersion in 3.5 wt.% NaCl solution at 20 °C (without applied stress) was −0.711 V vs. Saturated Calomel Electrode (SCE). During the early stage of exposure, minor fluctuations between −0.701 V and −0.722 V were recorded, indicating low-intensity corrosion occurring through microscopic defects in the oxide layer. During the 1500 h SCC test under constant tensile stress (σ = 0.8 R_p0.2_), the corrosion potential *E_corr_* of the PEO-coated samples remained stable within the range of −0.76 V to −0.81 V, showing no dynamic variations. This confirms that the coating did not undergo any sudden damage in any of the three tested specimens. All potentials in this study were measured against a Saturated Calomel Electrode (SCE, EK101), which is commonly used in electrochemical experiments simulating marine or chloride-rich environments.

**Figure 7:**
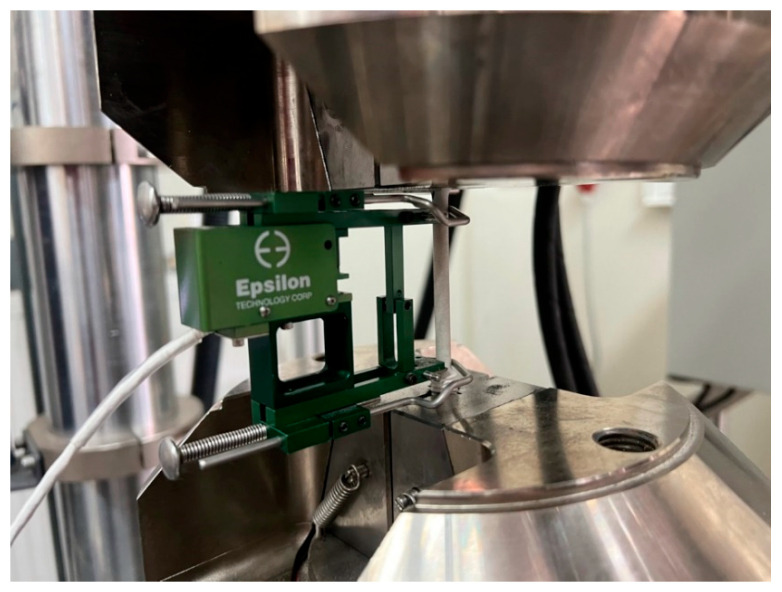
Al_2_O_3_ coated AW-5083 alloy sample after SCC testing in a universal testing machine with the extensometer Epsilon model 3542.

After SCC testing and corrosive exposure in NaCl solution were finished, coated AW-5083 alloy samples were cleansed, dried, and weighed. Afterwards, static tensile testing was performed, as prescribed by the PN-EN ISO 6892-1 standard [26] with a universal testing machine produced by Zwick&Roell type MPMD P10B [29] with an extensometer, Epsilon model 3542 [30] (Figure 7). The results were registered with testXpert II software version 3.61 released by Zwick&Roell.

## 3. Results

### 3.1. Results of the Potentiodynamic Testing

**Figure 8:**
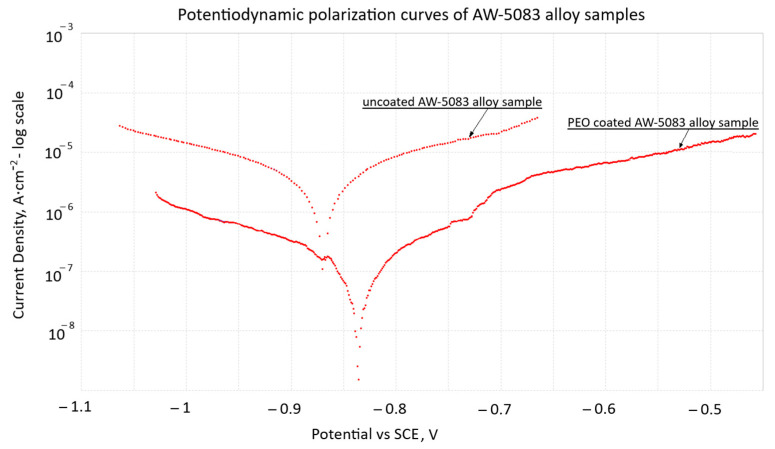
Potentiodynamic polarization curves (Tafel plots) of stress-free uncoated and PEO-coated AW-5083 alloy samples in 3.5 wt.% NaCl solution at 20 °C; potentials are referred to SCE (EK101).

The potentiodynamic polarization curves of uncoated and PEO-coated AW-5083 alloy samples tested in 3.5 wt.% NaCl solution at 20 °C are shown in Figure 8. For the uncoated alloy, both anodic and cathodic branches exhibit well-defined linear Tafel regions, which allowed the corrosion potential (*E_corr_*) and corrosion current density (*i_corr_*) to be determined using Metrohm’s NOVA software ver. 2.1.4 [31].

The corrosion current density measured for the PEO-coated sample (2.44 × 10^−7^ A·cm^−2^) is reduced by a factor of approximately 7 compared with the uncoated alloy (1.76 × 10^−6^ A·cm^−2^), confirming the markedly lower electrochemical activity and the improved barrier performance of the Al_2_O_3_ PEO layer. The coated sample also exhibits a slight positive shift of *E_corr_*, further indicating enhanced corrosion resistance in the chloride environment, which is consistent with the available results reported for other aluminum alloys coated with PEO layers [6].

These results confirm that the PEO coating effectively decreases charge-transfer kinetics and limits the anodic dissolution of the AW-5083 substrate under chloride exposure, which is consistent with trends previously reported for PEO coatings on aluminum alloys [6].

### 3.2. Results of the SCC Testing

The results of the static tensile testing of both PEO-coated and uncoated AW-5083 aluminum alloy samples after exposure to NaCl solution with and without stress corrosion cracking at the constant tensile stress value equal to 80% of R_p0.2_ are presented in Table 2. The values were averaged from three separate measurements of the samples of each kind.

**Figure 9:**
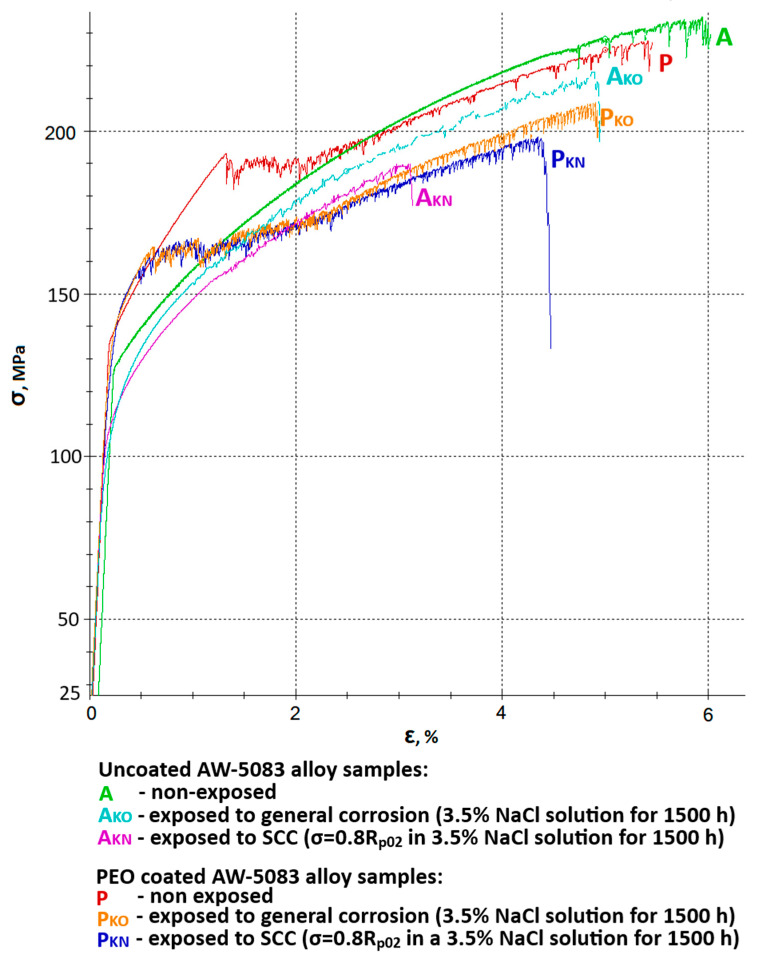
Stress–strain curves of AW-5083 aluminum alloy samples: A—uncoated samples, non-exposed, A_KO_—uncoated samples, exposed to general corrosion, A_KN—_uncoated samples, exposed to SCC, P—Al_2_O_3_ coated samples, non-exposed, P_KO_—Al_2_O_3_ coated samples, exposed to general corrosion, P_KN—_Al_2_O_3_ coated samples, exposed to SCC.

**Figure 10:**
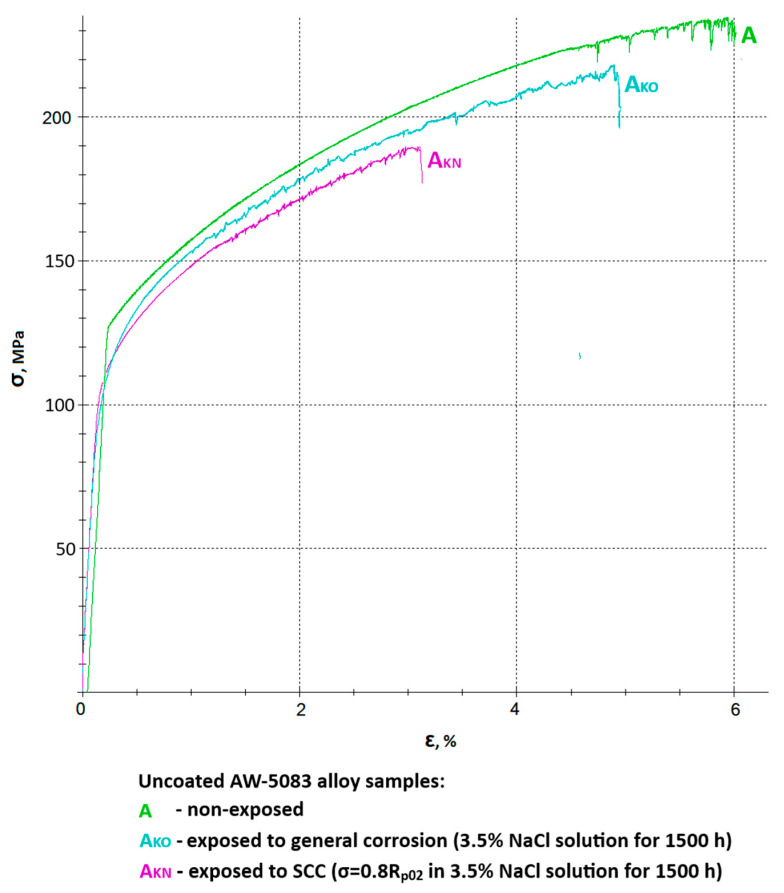
A set of stress–strain curves of the uncoated AW-5083 alloy samples: A—non-exposed, A_KO_—exposed to general corrosion, A_KN_—exposed to SCC (σ = 0.8 R_p0.2_).

**Figure 11:**
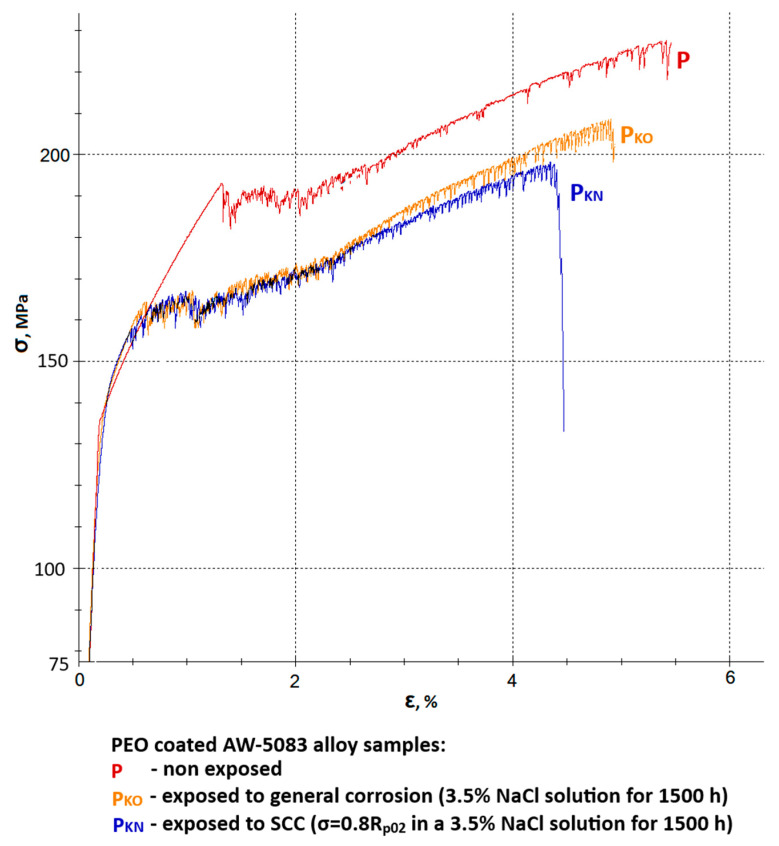
A set of stress–strain curves of the PEO coated AW-5083 alloy samples: P—non-exposed, P_KO_—exposed to general corrosion, P_KN_—exposed to SCC (σ = 0.8 R_p0.2_).

Figure 9 shows all stress–strain curves of AW-5083 aluminum alloy samples. In order to improve readability, it was divided into two separate figures—Figure 10 and Figure 11 show the same stress–strain curves of the uncoated and PEO coated samples, respectively.

Figure 10 shows a chosen set of stress–strain curves of the uncoated AW-5083 alloy samples. Mean Rm values were 234 MPa for uncoated, non-exposed samples, 218 MPa for uncoated samples exposed to general corrosion, and 190 MPa for uncoated samples exposed to SCC. These values present the scale of degradation of the unprotected materials in a corrosive environment. A mean strength reduction *K_Rm_* was calculated to be 6.8% (general corrosion) and 18.8% (SCC).

An even more pronounced degradation can be seen in respect to the plasticity of the material. Here, relative elongation A50 decreased from 5.6% to 4.7% for samples exposed to general corrosion and to 2.9% for samples exposed to SCC. A mean decrease in plasticity *K_A_* was equal to 16.1% and 48.2%, respectively. It ought to be noted that in the presence of chloride (Cl^−^) in the corrosive environment for 1500 h at the constant tensile stress value σ = 0.8 R_p0.2_ causes a significant (almost 50%) decrease in plasticity of the material. This fall is associated with multiple cracks on the surface prompted by the presence of stress and simultaneous aggressive chloride action [15].

Figure 11 presents a set of stress–strain curves of the PEO-coated AW-5083 alloy samples. Mean *R_m_* values were: 229 MPa for coated, non-exposed samples, 209 MPa for coated samples exposed to general corrosion and 199 MPa for coated samples exposed to SCC. These values show that the degradation rate of the coated alloy samples is notably lower compared to uncoated ones. A mean strength reduction *K_Rm_* was calculated to be 8.73% (general corrosion) and 13.1% (SCC). Moreover, degradation rate and scale are also lower in regard to plasticity of the material. Here, relative elongation *A*_50_ decreased from 5.3% to 5% for samples exposed to general corrosion and to 4.5% for samples exposed to SCC. Mean decrease in plasticity *K_A_* was equal to 5.7% and 15.1%, respectively. A corrosive environment with the presence of chloride (Cl^−^) causes a 15% decrease in the plasticity when a coated material is exposed for 1500 h at the constant tensile stress value σ = 0.8 R_p0.2_. This drop is significantly lower when compared to uncoated samples (48.2%). More than a three-fold lower decrease in plasticity of the alloy after coating may indicate that oxide PEO coating on this particular aluminum alloy has a relatively high strength.

The strength of the conversion coating with a specific share of aluminum polymorphs (α-Al_2_O_3_ i γ-Al_2_O_3_) in the case of AW-5083 alloy (medium strength alloy) is superior to the properties of a substrate [11]. This means that the stress level σ = 0.8 R_p0.2_ did not cause cracking on a substantial part of the surface exposed to NaCl solution, which resulted in significantly lower rate of plasticity degradation (15.1% coated vs. 48.2% uncoated). At this point, it would be natural to query how high strength alloys such as the 7XXX series alloys would behave after PEO coating when exposed to SCC (these alloys are characterized by R_p0.2_ values much higher than *R_m_* of the PEO coating itself).

The effectiveness of the PEO protective coating on the surface of the AW-5083 aluminum alloy was evaluated using the strength reduction factor *K_Rm_* (MPa) and the plasticity reduction facto *K_A_* (%) after exposure to a 3.5 wt.% NaCl solution for 1500 h at 20 °C. The reduction in the mechanical properties of the AW-5083 alloy samples was calculated according to Equations (1) and (2):

(1) $$K_{Rm}=\;\frac{1}{n}\;\Sigma \;\frac{R_{m}-R_{mk}}{R_{m}}100\%$$

(2) $$K_{A}\;=\;\frac{1}{n}\;\Sigma \;\frac{A_{50}-A_{k}}{A_{50}}$$

where

$R_{m}$—ultimate tensile strength before corrosive exposure (MPa);
$R_{\mathrm{mk}}$—ltimate tensile strength after corrosive exposure (MPa);
$A_{50}$—longation before corrosive exposure (%);
$A_{k}$—longation after corrosive exposure (%);
n—number of samples (3 of each kind).

The corrosion rate of the P_KN_ i P_KO_ samples was determined using the mass loss method, based on the change in sample mass over the exposure time, according to Equation (3). *V*_*kor*_—corrosion rate, expressed as the mass loss per unit surface area and exposure time (mg·mm^−2^·year^−1^):

(3) $$V_{kor}\;=\;\frac{M_{1}-M_{2}}{A\cdot t}$$

where

$M_{1}$—initial mass of the sample (g);
$M_{2}$—mass of the sample after corrosive exposure (g);
  A—active surface area of the sample (mm^2^);
t—corrosive exposure time (year, equivalent to 8760 h).

Statistical analysis was performed to evaluate differences between uncoated and PEO-coated AW-5083 alloy samples. All results are presented as mean ± standard deviation (SD), based on three independent measurements (n = 3). Statistical significance was assessed using a two-tailed Student’s *t*-test with unequal variances. Differences were considered statistically significant at *p* < 0.05.

**Figure 12:**
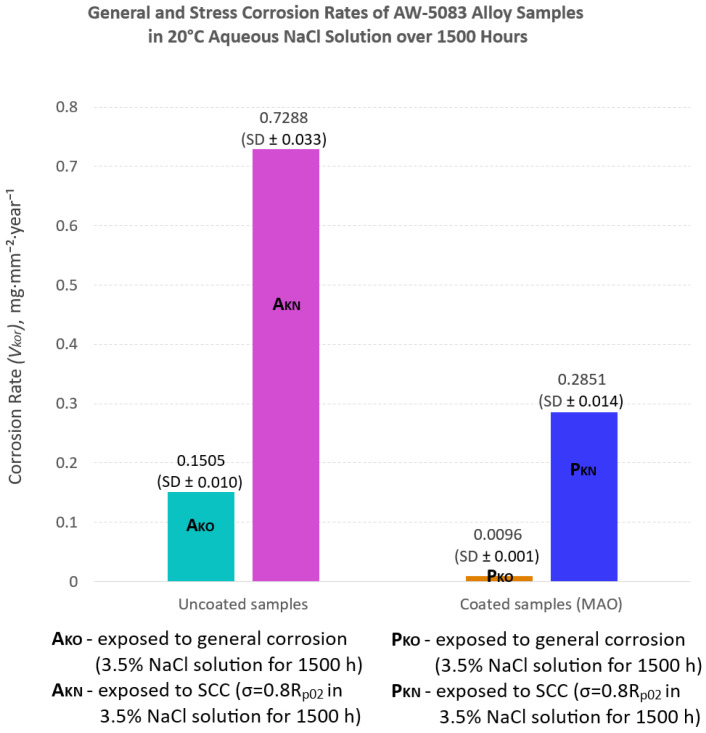
Corrosion rate (*V_kor_*) determined using the mass loss method for uncoated and PEO-coated AW-5083 alloy samples after exposure to 3.5 wt.% NaCl solution for 1500 h at 20 °C. The results are presented as mean ± SD (n = 3).

A comparison between corrosion rates for uncoated and PEO-coated AW-5083 alloy samples clearly indicates a significantly improved resistance to corrosion after surface modification of the alloy (Figure 12).

Figure 12 compares the corrosion rates of uncoated and PEO-coated AW-5083 alloy samples subjected to either general corrosion or SCC testing (expressed in milligrams per square millimeter per year, mg·mm^−2^·year^−1^).

For the uncoated AW-5083 alloy (A_KO_), the corrosion rate under general corrosion conditions was 0.1505 mg·mm^−2^·year^−1^, whereas for the coated samples (P_KO_) it decreased to 0.0096 mg·mm^−2^·year^−1^—a difference of approximately one order of magnitude. Thus, the uncoated alloy corroded about 15 times faster than the coated one. Under SCC testing conditions (σ = 0.8 R_p0.2_), the mean corrosion rate reached 0.7288 mg·mm^−2^·year^−1^ for the uncoated samples and 0.2851 mg·mm^−2^·year^−1^ for the PEO-coated specimens, representing a 2.6-fold reduction due to the presence of the oxide coating.

### 3.3. Mechanics of the PEO Coating Cracking

**Figure 13:**
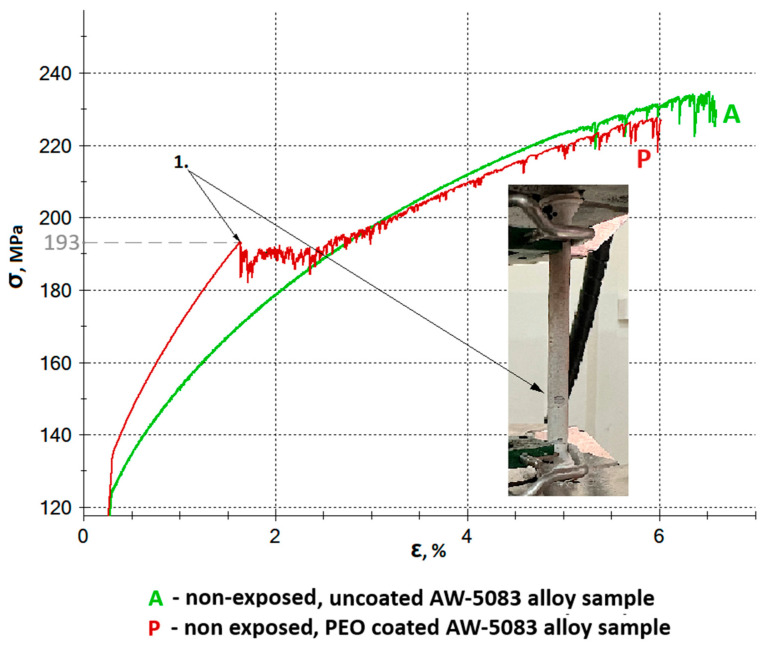
Engineering stress–strain curves of uncoated (A) and Al_2_O_3_ PEO-coated (P) AW-5083 alloy samples prior to corrosive exposure. A characteristic inflection point (“Point 1”) appears on the curve for the coated sample at approximately 193 MPa, corresponding to the initiation of microcracks in the oxide layer. The onset of coating cracking was recorded during the static tensile test (photograph on the right).

Figure 13 shows the enlarged sections of the stress–strain curves for the uncoated (A) and Al_2_O_3_-coated (P) AW-5083 alloy samples. The ultimate tensile strength *R_m_* of the uncoated alloy was 234 MPa, while for the Al_2_O_3_-coated samples it decreased slightly to 229 MPa–a reduction of 2.14% (see Table 2). This decrease results from the partial transformation of the aluminum substrate into aluminum oxide during the PEO process [32,33,34]. The coating consists partly of the durable α-Al_2_O_3_ polymorph [11,32], whose *R_m_* is approximately 260 MPa [27], and partly of the less stable γ-Al_2_O_3_ polymorph, with a tensile strength of about 60 MPa [27]. A characteristic inflection point was observed in the enlarged section of the stress–strain curve for the oxide-coated, non-exposed samples (curve P) at a stress value of approximately 193 MPa. This point (marked as “Point 1” in Figure 13) corresponds to the initiation of the first visible cracks in the Al_2_O_3_ coating. The onset of coating cracking was recorded during the static tensile test (see Figure 13).

**Figure 14:**
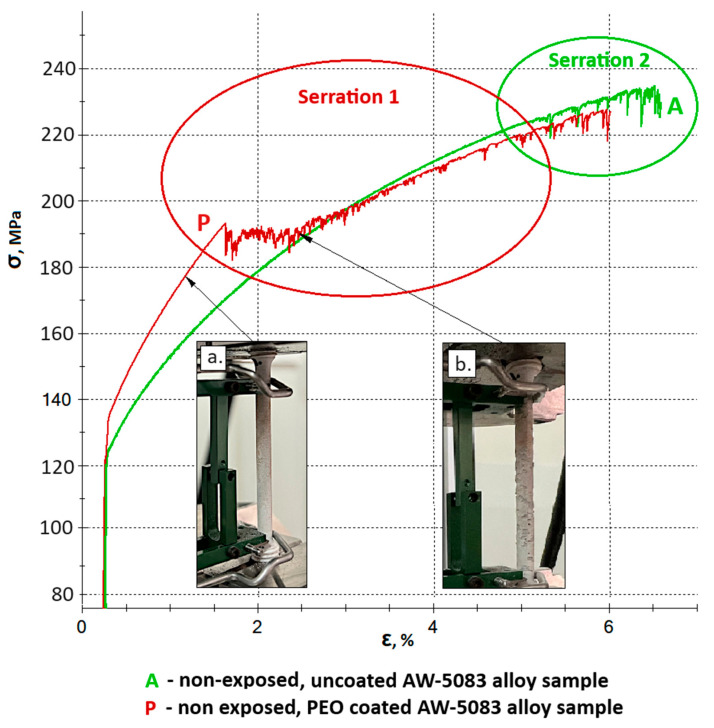
Serration 1 (associated with cracking and delamination of the oxide coating) marked on the stress–strain curve of the PEO-coated sample (P), and Serration 2 (physicochemical, related to microstructural changes in the metallic matrix) marked on the stress–strain curves of both PEO-coated (P) and uncoated (A) samples; (**a**) Sample P before the initiation of oxide coating cracks; (**b**) Sample P during propagation of visible cracks in the oxide coating.

Further serrations observed on the stress–strain curve of the coated sample (curve P) beyond Point 1 are associated with the propagation of cracks in the hard, strongly adherent Al_2_O_3_ PEO coating (Figure 14b). These fluctuations result from the gradual increase in tensile force during the static tensile test. The serrated profile remains visible until the oxide layer is completely detached from the sample surface. This behavior results from the measurement setup, as the extensometer arms were directly attached to the outer surface of the coating. As cracking progressed, the arms intermittently slipped over the fractured coating surface, producing dynamic variations in the recorded strain signal and appearing as serrations on the stress–strain curve.

The serration pattern observed on the P curve (identified as Serration 1 in Figure 14) should not be confused with the serrations caused by microstructural instabilities in the metallic matrix during tensile deformation, which are characteristic of certain aluminum alloys, including AW-5083 [35]. These latter instabilities, referred to as Serration 2 in Figure 14, have a completely different physical origin. The occurrence of coating-related serration does not exclude the presence of microstructural (physicochemical) serration–both phenomena may appear simultaneously and partially overlap. At this point, it should be clearly emphasized that the onset of cracking of the oxide coating formed on the AW-5083 alloy, accompanied by characteristic serrations, as well as the similar phenomenon observed in the uncoated AW-5083 alloy, occur at stress levels significantly exceeding the conventional yield strength R_p0.2_ of the base alloy, which is 137 MPa (Table 2), i.e., within the plastic deformation regime of the substrate, well above the elastic strain limit.

**Figure 15:**
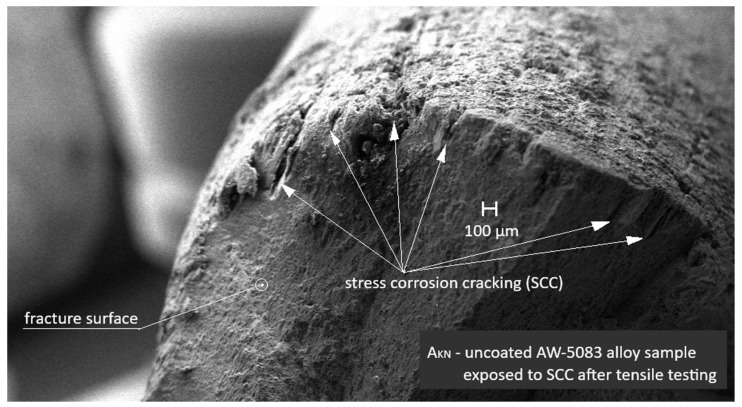
SEM image of the uncoated AW-5083 alloy sample after exposure to SCC (σ = 0.8 R_p0.2_) and subsequent tensile testing. Numerous SCC cracks are visible on the fracture surface.

Figure 15 shows an SEM image of the uncoated AW-5083 alloy sample exposed to stress corrosion cracking (SCC) under a tensile stress of σ = 0.8 R_p0.2_ in a 3.5 wt.% NaCl solution for 1500 h followed by tensile testing to fracture. Distinct stress corrosion cracks are visible on the fracture surface, indicating that SCC significantly reduced the ultimate tensile strength *R_m_* compared with the unexposed samples and those subjected only to general corrosion.

**Figure 16:**
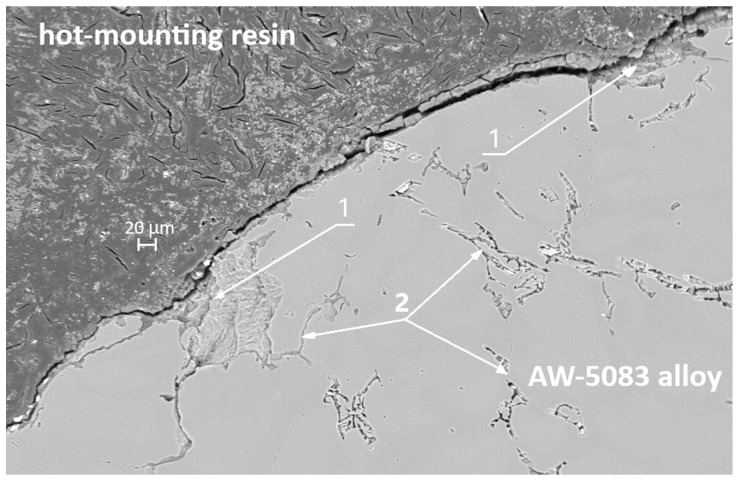
SEM micrograph of the cross-section of the AW-5083 alloy sample after stress corrosion cracking (A_KN_, σ = 0.8 R_p0.2_).

Figure 16 shows the SEM micrograph of the cross-section of the uncoated AW-5083 sample after stress-corrosion exposure. Regions marked with (1) indicate areas of severe material degradation localized along grain boundaries. The regions labeled (2) correspond to grain boundaries revealed after etching with Keller’s reagent. Their morphology is consistent with the locations where β-phase (Al_3_Mg_2_) precipitates are typically reported to form in sensitized Al–Mg alloys [15,36]; however, in the present study this association is qualitative only, as no local chemical or phase analysis (e.g., EDS or EBSD) was performed to confirm the presence of β-phase. The morphology of the regions marked (1)—their proximity to the sample surface (directly exposed to chloride ions), their open and branched geometry, and the strongly folded appearance of the adjacent metal—are characteristic features of samples subjected to stress-corrosion cracking. These features clearly indicate the synergistic action of corrosion and sustained tensile stress (σ = 0.8 R_p0.2_).

**Figure 17:**
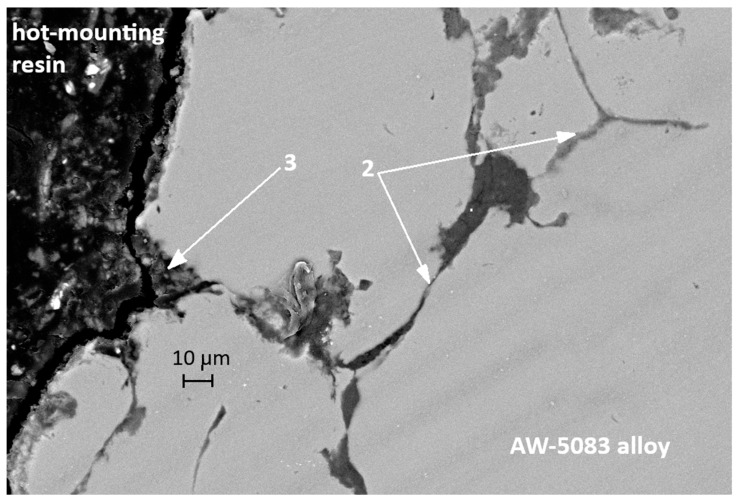
SEM micrograph of the cross-section of another the AW-5083 alloy sample after stress corrosion cracking (A_KN_, σ = 0.8 R_p0.2_).

Figure 17 also presents an SEM micrograph of the uncoated sample subjected to stress corrosion. The region marked with the number 3 shows a branched surface discontinuity located in the vicinity of features that are likely to coincide with grain boundaries of the alloy. Its morphology resembles crack-like damage often reported in stress-corroded Al–Mg alloys; however, based on the present SEM observations alone, it cannot be unambiguously classified as a fully developed intergranular crack. The dark area filled with the hot-mounting resin corresponds to a corrosion-induced cavity formed along the grain-boundary region. In the literature, such grain-boundary degradation is frequently attributed to the preferential dissolution of Mg-rich β-phase (Al_3_Mg_2_) in sensitized Al–Mg alloys [15,36]. In the present study, this interpretation remains qualitative only, as the presence of β-phase was not directly confirmed by local compositional or phase analysis. These fissures are also clearly visible in Figure 15, which shows an SEM image taken at a much lower magnification. 

**Figure 18:**
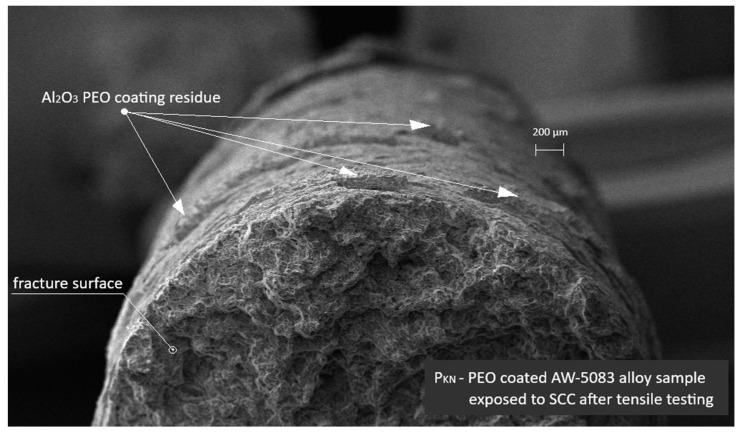
SEM image of the PEO-coated AW-5083 alloy sample after exposure to SCC (σ = 0.8 R_p0.2_) and tensile testing. Residual Al_2_O_3_ coating fragments are visible on the fracture surface.

Figure 18 presents an SEM image of the PEO-coated AW-5083 alloy sample tested under the same SCC conditions. Residual fragments of the Al_2_O_3_ PEO coating are visible on the fracture surface. The morphology of the fracture differs notably from that of the uncoated sample shown in Figure 15—the coating residue partially adheres to the substrate, suggesting a mitigating effect of the PEO layer on SCC propagation.

**Figure 19:**
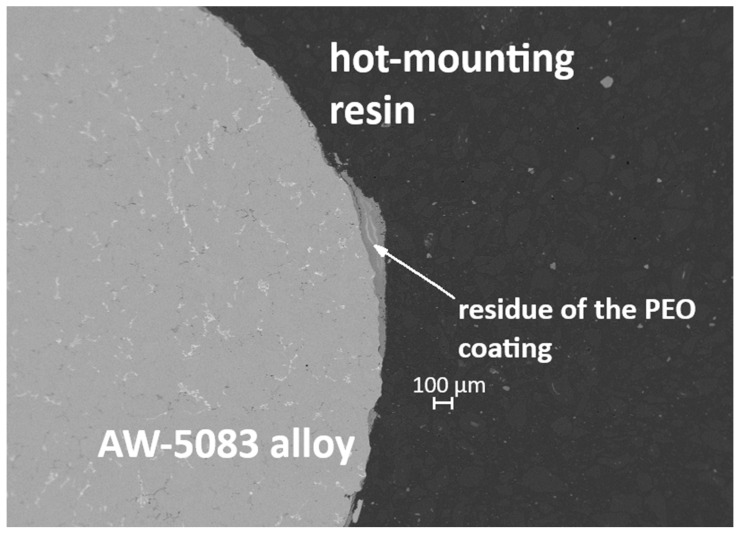
Cross-section of the PEO-coated specimen (PKN) after the static tensile test, showing cracked but still adherent remnants of the oxide layer.

**Figure 20:**
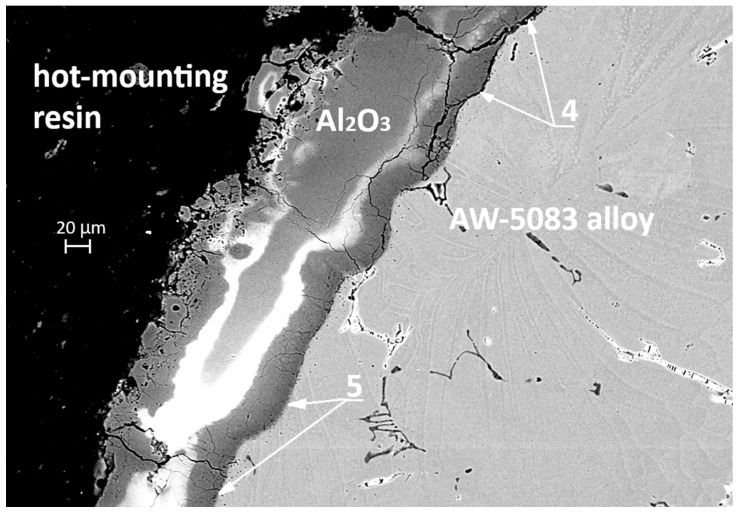
SEM cross-section of the PEO-coated specimen (PKN) after tensile testing, showing cracked yet strongly adherent oxide layer; areas of local delamination (4) and firmly bonded regions (5).

Figure 19 and Figure 20 present cross-sectional micrographs of specimens coated with the PEO layer (PKN) after the static tensile test. In both images, remnants of the oxide coating are visible, which—despite significant cracking induced by deformation—still adhere to the AW-5083 alloy substrate.

In Figure 20 (SEM, 500× magnification), a clear distinction can be observed between the areas where the coating has detached—marked with number 4—and the regions that remain strongly bonded to the substrate—marked with number 5. The pronounced cracking of the coating and its local delamination are a direct consequence of the stresses generated during the tensile test, whereas the preserved adhesion zones demonstrate the good bonding strength of the PEO coating to the alloy. Additionally, in Figure 20 it can be observed that the aluminum alloy surface in the PKN specimen did not undergo such severe degradation as the surface of the AKN specimen shown in Figure 16 and Figure 17 (without the PEO coating). This confirms that the PEO layer provides effective protection against the action of the chloride-containing solution.

## 4. Discussion

This study clearly demonstrates the significant influence of the PEO oxide coating on the corrosion resistance of the AW-5083 aluminum alloy. Unlike conventional anodizing, PEO produces a thick, partially crystalline, high-adhesion ceramic layer whose mechanical interaction with the substrate under tensile loading has not been widely reported in the literature for the AW-5083 alloy.

### 4.1. Polarization Curves

The Al_2_O_3_ layer produced by the PEO process on AW-5083 alloy forms a dense and strongly adherent barrier that effectively limits the access of chloride ions to the substrate. Although natural micropores are present, the compact structure of the inner oxide layer significantly restricts ionic transport at the coating–metal interface. As shown in Figure 8, the formation of the oxide layer results in a slight positive shift in the corrosion potential (*E_corr_*) and a substantial reduction in the corrosion current density (*i_corr_*).

Comparison of the polarization curves for the PEO-coated and uncoated samples reveals that the corrosion current density decreases from 1.76 × 10^−6^ A/cm^2^ for the uncoated alloy to 2.44 × 10^−7^ A/cm^2^ for the coated one—a reduction of approximately seven fold. This decrease reflects the reduced electrochemical activity of the substrate and the enhanced barrier properties provided by the PEO coating. At the same time, *E_corr_* shifts from −0.87 V to approximately −0.82 V, which is consistent with observations commonly reported for PEO coatings [37,38,39].

These effects are in line with the well-documented behavior of multi-layered alumina coatings produced by PEO, consisting of a dense inner barrier layer and a more porous outer layer. Even though such coatings are not completely impermeable, their architecture substantially limits charge-transfer processes at the coating–electrolyte interface, thereby improving corrosion resistance. The protective performance of PEO coatings is known to depend on processing parameters.

It should also be noted that the present study was focused primarily on SCC behavior under mechanical loading, and therefore the electrochemical characterization was limited to potentiodynamic measurements without full impedance spectroscopy.

### 4.2. Mechanical Behavior and Stress Corrosion Cracking (SCC)

The uncoated samples showed pronounced susceptibility to chloride-induced corrosion. After 1500 h of exposure to 3.5 wt.% NaCl solution, the ultimate tensile strength (*R_m_*) of the alloy decreased by 6.8%, while its plasticity (elongation *A*_50_) was reduced by 16.1%. Under SCC conditions, with a constant tensile stress of 80% of R_p0.2_, the degradation of the uncoated alloy became even more severe, resulting in an 18.8% drop in *R_m_* and a nearly 50% reduction in *A*_50_ (Table 3 and Figure 12). Such a drastic decline in mechanical properties reflects the synergistic effect of tensile stress and an aggressive chloride environment, which accelerates crack initiation and propagation [36,40]. The magnitude of these reductions is in line with previously reported SCC behavior of Al–Mg alloys, in which intergranular cracking driven by β-phase dissolution and stress localization has been shown to lead to accelerated mechanical degradation under chloride exposure [36].

The application of the PEO coating effectively mitigated these adverse effects. The PEO-coated samples demonstrated substantially higher resistance to corrosion. Under general corrosion conditions, the corrosion rate was 0.0096 mg·mm^−2^·year^−1^, approximately 15 times lower than that of the uncoated material (0.1505 mg·mm^−2^·year^−1^). Under SCC conditions, the corrosion rate decreased by a factor of 2.6 (from 0.7288 to 0.2851 mg·mm^−2^·year^−1^). Similarly, the loss of plasticity in the corrosive environment was three times smaller for the PEO-coated samples (15.1%) than for the uncoated ones (48.2%) (Table 3, Figure 12).

In SCC, crack initiation often occurs at local surface defects or chemically heterogeneous regions [41]. The PEO coating adheres strongly to the substrate–for the AW-5083 alloy, cracking and partial detachment begin at a stress of approximately 190 MPa (Figure 13)—which likely reduces the probability of such defect formation, thereby suppressing crevice and pitting corrosion and delaying SCC initiation [10]. Although the present work did not include a full microscale or nanoscale structural characterization of the coating during deformation, the interpretation of coating cracking and partial detachment is supported by fractographic and cross-sectional SEM observations (Figure 15, Figure 16, Figure 17, Figure 19 and Figure 20). These images reveal localized coating rupture and partial decohesion at stresses corresponding to the onset of Serration 1 in the mechanical curve, confirming the mechanical origin of the first serration event. Such morphology is consistent with previously reported deformation mechanisms for oxide-coated aluminum alloys, where brittle surface layers accommodate strain through microcracking without compromising the substrate’s load-bearing capacity [11,41].

Since the surface of the PEO coating exhibited a relatively high roughness (Ra = 4.171 μm and Rz = 20.756 μm), and the samples were not polished after coating, the authors were particularly interested in determining whether, and to what extent, this surface morphology could reduce the tensile strength (*R_m_*) compared with the uncoated samples.

The mechanical response of the PEO-coated samples during tensile testing (Figure 13 and Figure 14) provides additional insight into the deformation process. The occurrence of Serration 1 on the stress–strain curve corresponds to localized cracking of the oxide coating, whereas Serration 2 reflects the intrinsic dynamic strain aging behavior of the AW-5083 alloy matrix. The two effects may coexist but act independently: coating cracking is limited to the surface layer and does not compromise the load-bearing capacity of the substrate. Instead, the partial cracking of the PEO layer allows strain accommodation and prevents sudden delamination. This mechanical interaction between the coating and the ductile substrate explains why the PEO-coated alloy retains its overall strength and ductility even after prolonged exposure to SCC conditions. It is important to note that the present investigation primarily focused on macroscopic corrosion behavior and mechanical response under SCC conditions. While the SEM-based observations provided valuable qualitative insight into the cracking morphology, a more advanced structural characterization–such as EBSD, TEM, or FIB cross-sectioning–could further elucidate the micro- and nanoscale mechanisms governing coating deformation and crack propagation. Nevertheless, the combined electrochemical, mechanical, and microstructural evidence presented in this study establishes a coherent interpretation of the protective role of the PEO coating and its influence on SCC susceptibility.

## 5. Conclusions

This study demonstrates that the Al_2_O_3_ PEO coating is an effective way to enhance the corrosion and SCC resistance of the AW-5083 aluminum alloy. The coating not only drastically reduces the general corrosion rate but also provides significant protection against the combined action of tensile stress and a corrosive environment, which in the uncoated material leads to severe loss of plasticity. The results show that the mechanical properties and strength of the PEO coating are superior to those of the substrate, enabling effective protection against plasticity loss under SCC conditions. The findings have considerable practical significance: PEO technology can be used to enhance the SCC resistance of AW-5083 alloy components, particularly in aggressive environments such as seawater. Improving the service life and reliability of aluminum structures–for instance in shipbuilding and the energy sector–may yield substantial economic and environmental benefits.

### Future Directions

Moreover, this study provides new insight into how PEO coatings interact mechanically with the AW-5083 substrate under combined electrochemical and mechanical loading. The results clarify the role of the coating in suppressing the initiation of corrosion-induced microdefects that typically trigger SCC in Al–Mg alloys, thereby offering a mechanistic explanation for the enhanced resistance observed in the coated samples. Future research should focus on:

- A detailed analysis of the PEO coating microstructure after SCC exposure to identify damage sites and crack propagation mechanisms within the coating;
- Optimization of PEO process parameters (electrolyte composition, current density, oxidation time) to further enhance the coating’s protective performance;
- Long-term SCC testing under variable environmental and stress conditions to fully assess coating durability;
- Investigation of the PEO coating’s influence on other forms of local corrosion under stress, such as corrosion fatigue;
- Studies of PEO coatings on the AW-5083 alloy after corrosive exposure, including SCC, using electrochemical impedance spectroscopy (EIS).

The authors are particularly interested in the application of PEO-coated elements in marine environments–specifically on open-deck machinery and equipment. Considering the high corrosion and wear resistance of PEO coatings compared to the substrate material [10], this technology is planned to be applied to transport rollers and sliding components used on aluminum or composite vessels, where mass reduction is critical.

In addition, studying PEO coatings on high-strength aluminum alloys (e.g., 7XXX series), whose R_p0.2_ values exceed the ultimate tensile strength of the coating itself, would provide valuable insights into the mechanics of coating cracking and its limits of applicability.

In conclusion, the use of Plasma Electrolytic Oxidation Al_2_O_3_ coatings on AW-5083 alloy represents a promising approach for mitigating stress corrosion cracking. The present research contributes to the understanding of surface protection mechanisms and offers a foundation for the further development of aluminum alloys with enhanced resistance to degradation in demanding service environments.

## Figures and Tables

**Table 1:** Chemical composition of the cold-rolled aluminum alloy AW-5083 sheet.

Alloy	Chemical Composition [%]										Declaration of Conformity	Norm
5083	Mg	Mn	Fe	Si	Cu	Cr	Zn	Ti	Ga	Al	1141807/55125/ 2018	EN AW-5083
	4.27	0.31	0.35	0.28	0.04	0.06	0.01	0.02	0.03	the rest		

**Table 2:** The results of tensile testing of the AW-5083 aluminum alloy samples (data presented as mean ± SD; n = 3 for each sample type).

Alloy	Corrosion Type	Sample Group	E [GPa]	R_p0.2_ [MPa]	*R_m_* [MPa]	*A*_50_ [%]	Change in Mass Due to Corrosive Factors
AW-5083	Mechanical properties of the samples that have not been exposed to corrosive factors						
	n/a	A—uncoated	70 ± 0	137 ± 2	234 ± 3	5.6 ± 0.1	n/a
		P—PEO-coated	71 ± 0	146 ± 3	229 ± 3	5.3 ± 0.1	n/a
	Mechanical properties of the uncoated samples after exposure to 3.5 wt.% NaCl solution for t = 1500 h						
	SCC σ = 0.8 R_p0.2_	A_KN_	68 ± 1	122 ± 4	190 ± 4	2.9 ± 0.2	−0.0914 ± 0.0042 g
	General corrosion σ = 0	A_KO_	69 ± 0	124 ± 3	218 ± 3	4.7 ± 0.2	−0.0189 ± 0.0012 g
	Mechanical properties of the PEO coated samples after exposure to 3.5 wt.% NaCl solution for t = 1500 h						
	SCC σ = 0.8 R_p0.2_	P_KN_	68 ± 1	146 ± 3	199 ± 4	4.5 ± 0.2	−0.0358 ± 0.0018 g
	General corrosion σ = 0	P_KO_	69 ± 0	146 ± 2	209 ± 3	5.0 ± 0.1	−0.0012 ± 0.0001 g

**Table 3:** Mechanical properties and corrosion resistance of AW-5083 aluminum alloy samples–uncoated and Al_2_O_3_ PEO-coated–before and after corrosive exposure.

Material (Alloy) and Coating	Mechanical Properties Before Corrosive Exposure			Mechanical Properties After Corrosive Exposure in 3.5 wt.% NaCl Solution for 1500 h at 20 °C											
				General Corrosion σ = 0						Stress Corrosion Cracking at the Constant Tensile Stress Value Equal to 80% of R_p0.2_					
	*R_m_* [MPa]	R_p0.2_ [MPa]	*A*_50_ [%]	*R_m_* [MPa]	R_p0.2_ [MPa]	*A*_50_ [%]	*K_Rm_* [%]	*K_A_* [%]	*V_kor_* [mg·mm^−2^·year^−1^]	*R_m_* [MPa]	R_p0.2_ [MPa]	*A*_50_ [%]	*K_Rm_* [%]	*K_A_* [%]	*V_kor_* [mg·mm^−2^·year^−1^]
AW-5083 PEO-coated	229 ± 3	146 ± 3	5.3 ± 0.1	209 ± 2	153 ± 2	5 ± 0.1	5 ± 0.1	8.73	5.7	0.0096 ± 0.001	199 ± 2	153 ± 1.5	4.5 ± 0.1	13.1	15.1	0.2851 ± 0.014
AW-5083 uncoated	234 ± 3	137 ± 3	5.6 ± 0.1	218 ± 3	124 ± 3	4.7 ± 0.1	6.8	16.1	0.1505 ± 0.010	190 ± 3.5	122 ± 3.3	2.9 ± 0.1	18.8	48.2	0.7288 ± 0.033

## Data Availability

The original contributions presented in this study are included in the article. Further inquiries can be directed to the corresponding author.

## Abbreviations

The following abbreviations are used in this manuscript:

SCC	Stress Corrosion Cracking
PEO	Plasma Electrolytic Oxidation
